# Phase-targeted X-ray diffraction

**DOI:** 10.1107/S1600576716011936

**Published:** 2016-09-01

**Authors:** G. M. Hansford

**Affiliations:** a University of Leicester, Space Research Centre, Department of Physics and Astronomy, Leicester LE1 7RH, UK

**Keywords:** energy-dispersive X-ray diffraction, phase specificity, target phases, handheld instrumentation, retained austenite, in-line instrumentation

## Abstract

A powder X-ray diffraction (XRD) method to enhance the signal of a specific crystalline phase within a mixture is presented for the first time. Specificity to the targeted phase relies on finding coincidences in the ratios of crystal *d* spacings and the ratios of elemental characteristic X-ray energies. Such coincidences can be exploited so that the two crystal planes diffract through the same scattering angle at two different X-ray energies. An energy-resolving detector placed at the appropriate scattering angle will detect a significantly enhanced signal at these energies if the target mineral or phase is present in the sample. When implemented using high scattering angles, for example 2θ > 150°, the method is tolerant to sample morphology and distance on the scale of ∼2 mm. The principle of the method is demonstrated experimentally using Pd *L*α_1_ and Pd *L*β_1_ emission lines to enhance the diffraction signal of quartz. Both a pure quartz powder pellet and an unprepared mudstone rock specimen are used to test and develop the phase-targeted method. The technique is further demonstrated in the sensitive detection of retained austenite in steel samples using a combination of In *L*β_1_ and Ti *K*β emission lines. For both these examples it is also shown how the use of an attenuating foil, with an absorption edge close to and above the higher-energy characteristic X-ray line, can serve to isolate to some degree the coincidence signals from other fluorescence and diffraction peaks in the detected spectrum. The phase-targeted XRD technique is suitable for implementation using low-cost off-the-shelf components in a handheld or in-line instrument format.

## Abstract 2

A method to enhance the X-ray diffraction signal of a specific targeted crystalline phase within a sample is presented. This technique can be implemented in a handheld or in-line instrument format.

## 1. Introduction   

For many applications in research and commerce, X-ray diffraction (XRD) represents the gold-standard materials analysis technique because of the uniqueness of the diffraction pattern of each distinct microcrystalline phase, based on the set of *d* spacings that are specific to each crystal structure. Increasingly, XRD is also applicable to semi-crystalline phases, nanomaterials and even amorphous phases [see, for example, Chipera & Bish (2013 ▸), Murthy & Minor (1990 ▸), Gates *et al.* (2014 ▸) and Pu *et al.* (2008 ▸)]. The vast majority of applications are served by laboratory diffractometers or synchrotron facilities that require the sample to be brought to the instrument and, usually, also require careful and destructive preparation of the sample. A handful of portable XRD instruments that can be taken to the sample and applied non­destructively have been developed, mainly for archaeometry research (Uda, 2004 ▸; Uda *et al.*, 2005 ▸; Cuevas & Gravie, 2011 ▸). The Duetto instrument (Sarrazin *et al.*, 2008 ▸; Chao *et al.*, 2014 ▸) was developed for this purpose using technology derived from the NASA Mars CheMin instrument (Bish *et al.*, 2014 ▸, and references therein). To date, only one field-portable instrument is available commercially (Sarrazin *et al.*, 2008 ▸; Chipera *et al.*, 2009 ▸), mainly intended for geological and mining applications. Although XRD instrumentation is widely used for industrial process control, these instruments are rarely employed in-line because of the difficulty of automating sample preparation and data analysis (O’Dwyer & Tickner, 2008 ▸; Dhanjal *et al.*, 2006 ▸; Anderson *et al.*, 2015 ▸).

In this paper, a method to enhance the XRD signal from a specific targeted phase within a mixture is presented for the first time. The technique is suitable for implementation in a handheld or in-line instrument format. In general terms, potential applications are those in which a specific phase or mineral is key for the intended use of the sample material. This specific phase may be crucial to the function of the material, or it could be an important and undesirable impurity. The technique could be used to determine either the presence or absence (more precisely, an upper limit) of a particular phase, or whether a material passes a threshold criterion in quality control applications. As an example, retained austenite is often a key phase in the manufacture of different types of steel. Austenite is a high-temperature allotrope of iron or iron alloy that allows higher levels of carbon to be dissolved. The amount of austenite retained in the microstructure after cooling is an important factor in determining some of the strength characteristics of the steel. Depending on the intended use of any given steel, retained austenite may be either advantageous or detrimental to the mechanical properties (Herring, 2005 ▸). For example, the tool and die industry requires minimum amounts of retained austenite and higher levels of ferrite and martensite, because maximum hardness and resistance to wear are usually crucial in these applications and austenite reduces the attainable hardness. Conversely, some retained austenite is beneficial in the bearing and gear industry because it suppresses crack propagation.

Importantly, the proposed technique avoids the need for an expensive and bulky goniometer, and it can be implemented in a fixed geometry using low-cost technology; see §2 for details. It is necessary to tailor some details of the instrument design to the targeted phase. The technique is a powder XRD method, appropriate for microcrystalline samples, and is closely analogous to energy-dispersive XRD (EDXRD) in its implementation, even though the source continuum spectrum is not utilized. The rationale underlying the phase-targeted XRD method is that it enables a relatively simple low-cost instrument design with no moving parts, and avoids the need for any sample preparation. The sensitivity of the method is not expected to compete with conventional floor-mounted and benchtop diffractometers, so the key advantages lie in the capability of making rapid, *in situ* and nondestructive measurements. Consequently, the primary applications are likely to be commercial, although applications in the cultural heritage field are feasible.

## 2. Theory   

### 2.1. Method principle   

Phase specificity in the new XRD technique relies on finding coincidences in the ratios of crystal *d* spacings and the ratios of elemental characteristic X-ray energies. Such co­incidences can be exploited so that the two crystal planes diffract through the same scattering angle at two different X-ray energies. An energy-resolving detector placed at the appropriate scattering angle will detect a significantly enhanced signal at these energies if the target mineral or phase is present in the sample.

**Figure 1:**
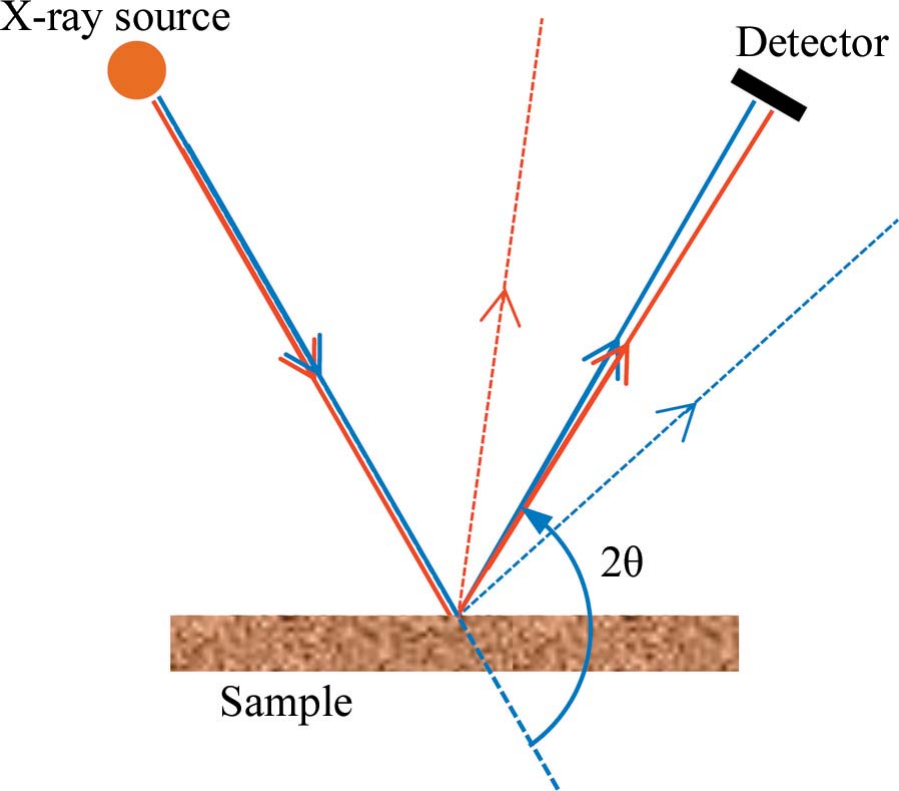
A schematic diagram to illustrate the basic principle of phase-targeted XRD. Two rays from the source, representing different X-ray energies, are diffracted through approximately the same angle to reach an energy-resolving detector positioned to intersect the diffracted rays.

The principle of the method can be readily described using the Bragg law for diffraction of X-rays from crystal planes expressed in the energy domain: *Ed* sin θ = 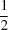 *hc*, where *E* is the photon energy, *d* is the crystal *d* spacing, 2θ is the scattering angle, *h* is Planck’s constant and *c* is the speed of light in a vacuum. Considering the diffraction of two different X-ray energies from two different crystal *d* spacings,
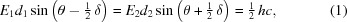
where δ must be small (the coincidence criterion), *E*_1_ and *E*_2_ are the characteristic energies emitted simultaneously by the X-ray source, and *d*_1_ and *d*_2_ are the *d* spacings of the target phase. Cast in this form, 2θ is the average scattering angle of the two reflections, δ is the difference between each reflection angle and the average, and 2δ gives the total angular mismatch in 2θ. Rearranging equation (1) gives
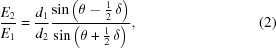
showing that the ratio of the characteristic energies must closely match the inverse ratio of the *d* spacings. The principle of the method is illustrated in schematic form in Fig. 1 ▸. Each of the *d* spacings must also satisfy
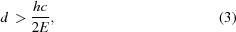
which is the energy-domain equivalent of the diffraction requirement *d* > 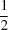
λ, where λ is the X-ray wavelength.

Implementation of this coincidence XRD method must accommodate the finite angular mismatch 2δ between the two reflections, and any practical setup will in any case encompass a range of 2θ scattering angles. In this regard, it is instructive to consider the dependence of *d*-spacing discrimination on the 2θ scattering angle. Equation (1) can be re-cast in the following form:
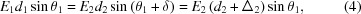
where θ_1_ = θ − 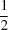
δ, and Δ_2_ is the mismatch between *d*_2_ and the ideal value that would bring the two scattering angles into exact coincidence. In the right-hand side of this equation, the mismatch in the coincidence has been assigned to the *d* spacing rather than to the scattering angle. Rearranging equation (4) gives
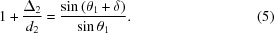
Expanding the sine sum term, applying small-angle trigonometric approximations, eliminating terms of order δ^2^ and higher, and applying the approximation tan θ_1_ ≃ tan θ gives the following simple result:
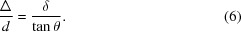
The subscripts have been dropped from the terms in this equation to generalize it. Equation (6) relates the angular mismatch of a coincidence to the equivalent *d*-spacing mismatch, and shows that the fractional *d*-spacing mismatch is much smaller at high scattering angles for any given angular mismatch. In other words, *d*-spacing discrimination is much better in a back-reflection geometry and specificity to the target phase is improved. Fortunately, there are other reasons why high-angle coincidences are advantageous. For any given detector size, a greater arc length of the Debye rings will be captured, which improves sensitivity and reduces the effects of particle statistics. A back-reflection geometry also yields improved tolerance to sample morphology and relaxed constraints on the precise positioning of the sample (see §4.1). This benefit works in much the same way as in back-reflection EDXRD (Hansford, 2011 ▸, 2013 ▸), although the phase-targeted XRD technique does not have the extreme insensitivity to sample morphology engendered by back-reflection EDXRD. For the normal range of *d* spacings accessed in typical powder diffraction setups, for example 1–10 Å, high scattering angles imply low X-ray energies, up to a few kiloelectronvolts (Hansford, 2011 ▸). Lastly, this geometry is also favourable for a compact and lightweight instrument design.

The fact that the coincidence criterion requires a specific value for the *d*-spacing ratio, rather than the *d* spacings themselves, has some subtle consequences. For example, the same coincidence may be applicable across a range of solid solution phases, as long as the crystal symmetry is unchanged and equation (3) is satisfied. Depending on the details of the structural changes and on the chosen reflections, *d*-spacing ratios may or may not remain the same across a solid solution. In any case, changes in the individual *d* spacings themselves will alter the scattering angle of the coincidence.

In general terms, it is preferable for the reflection indices corresponding to the two *d* spacings to involve all of the unique unit-cell dimensions of the crystal structure. For example, for a mineral belonging to either the tetragonal or the hexagonal crystal system, it would be a disadvantage if both *d* spacings corresponded to reflections of the form *hk*0, because the *c* dimension of the unit cell then becomes irrelevant and specificity is reduced, at least in principle. A second crystalline phase that has a sufficiently similar *a* unit-cell dimension to the target phase, and belongs to the same crystal system, could give an interfering coincidence signal even if the *c* dimension were different. These considerations are especially interesting for phases belonging to the cubic crystal system, for which *d* spacings are given by *d* = *a*/(*h* ^2^ + *k* ^2^ + *l* ^2^)^1/2^, where *a* is the unit-cell dimension and *h*, *k* and *l* are the Miller indices of the planes. In this case, the ratio of the *d* spacings corresponding to any two reflections is independent of the unit-cell size. A coincidence that has been found for one cubic phase applies to all cubic phases as long as equation (3) is also satisfied, although one or both of the reflections may be systematically absent owing to general or special reflection conditions. Even for reflections that are not excluded by symmetry, the intensities may vary significantly from one phase to another because of the structure factor, so a co­incidence that is suitable for one cubic phase may not represent a good solution for another. The unit-cell size determines the scattering angle at which the coincidence occurs, and this factor is another one that may count against a particular coincidence for any given cubic phase.

### 2.2. Additional constraints   

A software program has been written to compare the ratios of target phase *d* spacings with the ratios of characteristic X-ray energies from a selected range of elemental *K*- and *L*-series emission lines. Coincidences that satisfy user-specified limits for the angular mismatch and a lower bound for the scattering angle are identified. Application of the software demonstrates that it is not difficult, in general, to find co­incidences that satisfy reasonable angular criteria. However, there are several other constraints and trade-offs that must be taken into account when selecting the best coincidences. To achieve reasonable sensitivity, the X-ray emission lines and diffraction intensities corresponding to the selected *d* spacings should be relatively strong. The enhanced diffraction peaks should not lie close to any fluorescence peaks likely to be present in the detected spectrum and, for the same reason, the element used to generate the characteristic emission lines should not be present in the sample at higher than trace levels. The source element should also not be excessively expensive, difficult to handle or unstable. The requirement to search for suitable coincidences and select a source element and scattering angle means that the design and setup of an instrument that implements the phase-targeted XRD method must be tailored to the target phase.

In selecting the *d* spacings of the target phase or mineral for the coincidence method, it is also useful to take into account the target application. In most applications, the other phases that are likely to be present in the sample will be known in advance. To avoid the possibility of a false positive signal, the chosen target *d* spacings should not lie too close to the *d* spacings of any of the other phases present. Equation (6) can be used to calculate the *d*-spacing differences required to avoid interferences.

There are several ways in which the coincidence technique described thus far can be extended. More extensive searches for accurate high-intensity and high-angle coincidences can be performed by alloying the source elements to increase the number of characteristic X-ray energy ratios available (an example is given in §2.3). The relative amounts of the two elements in the alloy can be tailored in order to equalize the intensity of the two coincidence diffraction lines and therefore optimize the sensitivity. Improved specificity can be achieved by exploiting three-line (or higher) coincidences, although in general this implementation leads to reduced sensitivity because the weakest of the enhanced diffraction peaks will limit the sensitivity. Another possibility is the simultaneous enhanced detection of two different phases by selecting one *d* spacing of each phase for the coincidence. In this case it is probably essential that the intended application guarantees specificity. Further possibilities are opened up if more than one source or detector are employed in the instrument.

### 2.3. Example coincidences   

There are several potential applications in the targeted detection and quantification of quartz, such as the protection of mine workers’ health from the effects of respirable silica (Cauda *et al.*, 2016 ▸). Table 1 ▸ shows two favourable co­incidences that can be used to enhance the detection of quartz, employing either Pd or Ce as the source element. The Pd coincidence has characteristic emission lines that are quite closely spaced, but off-the-shelf energy-resolving detectors can nevertheless resolve these energies sufficiently well. The scattering angle is relatively high at 2θ = 154.5° and the total angular mismatch of 2δ = 1.1° is reasonable. In fact, there is a close third line due to the enhancement of quartz 111 by Pd *L*α_2_ (2833.7 eV) at 2θ = 156.0° [all characteristic and absorption-edge X-ray energies in this paper are taken from Deslattes *et al.* (2003 ▸)]. The *L*α_2_ emission line only has approximately one-tenth of the intensity of *L*α_1_ and the two lines are not spectroscopically resolved, so this enhancement has minor significance. By comparison, the Ce coincidence occurs at a higher angle and is a more accurate match, but has lower enhanced intensities and therefore lower sensitivity. The *d* spacings for the Ce coincidence are significantly smaller than those for the Pd coincidence, which is disadvantageous because the average density of crystal *d* spacings increases towards lower *d* spacings for all phases (Buchsteiner & Stüßer, 2009 ▸; Hansford, 2011 ▸), leading to greater potential for interferences. This factor, along with the higher expected intensities, leads to the choice of the Pd coincidence as the best one.

A second example is the enhanced detection of retained austenite in steels. Austenite is a high-symmetry (cubic) phase with a small unit cell and therefore has a relatively sparse set of *d* spacings. Consequently, it has not been possible to identify any coincidences using individual source elements that are free from major disadvantages, such as overlap with the fluorescence peaks of elements frequently present in steels. However, by considering pairs of source elements, as described in §2.2, a suitable coincidence has been found and is shown in Table 1 ▸. It involves an *L*-series emission line of In and the Ti *K*β line, leading to a very accurate high-angle coincidence. The relevant *d* spacings of austenite are not close to any *d* spacings of ferrite (α-Fe) or martensite. This co­incidence may not be suitable for the relatively small proportion of steels that contain Ti.

A complicating factor for austenite is that the presence of alloying elements affects the unit-cell dimensions and therefore the *d* spacings. As described in §2.1, for a cubic phase all the *d* spacings vary proportionately for a change in the unit-cell size, so that the coincidence remains valid but the scattering angle changes. Considering just the carbon content of a steel, the variation of the unit-cell dimension is given by *a* = 3.5780 + 0.033*x* Å, where *x* is the wt% of carbon (Dyson & Holmes, 1970 ▸). Assuming a carbon content ranging from zero up to a maximum of 2.1 wt%, the scattering angle will vary between 2θ = 167.1 and 154.2°. Most other alloying elements also increase the unit-cell size and consequently equation (3) will not, in general, be violated. An imaging X-ray detector subtending this angular range could serve to detect and quantify the amount of retained austenite in a wide range of steel samples. The unit-cell dimension of austenite is also affected by residual stress, but to a significantly smaller extent than the influence of carbon content [see, for example, Streicher-Clarke *et al.* (2005 ▸)]. The effect on the 2θ angle of the coincidence in Table 1 ▸ is expected to be less than 1°.

As already noted, austenite is a cubic phase, so the co­incidence shown in Table 1 ▸ is applicable to other cubic phases, as long as equation (3) is satisfied and neither of the 200 or 220 reflections is excluded by reflection conditions. In particular, austenites of other alloys and metals that crystallize in the face-centred cubic structure can be sensitively detected using the In–Ti coincidence. Examples include superalloys, and aluminium and its alloys. Body-centred cubic metals cannot be detected using this coincidence because the unit cells are considerably smaller and equation (3) is violated.

### 2.4. Enhancement by foil absorption   

**Figure 2:**
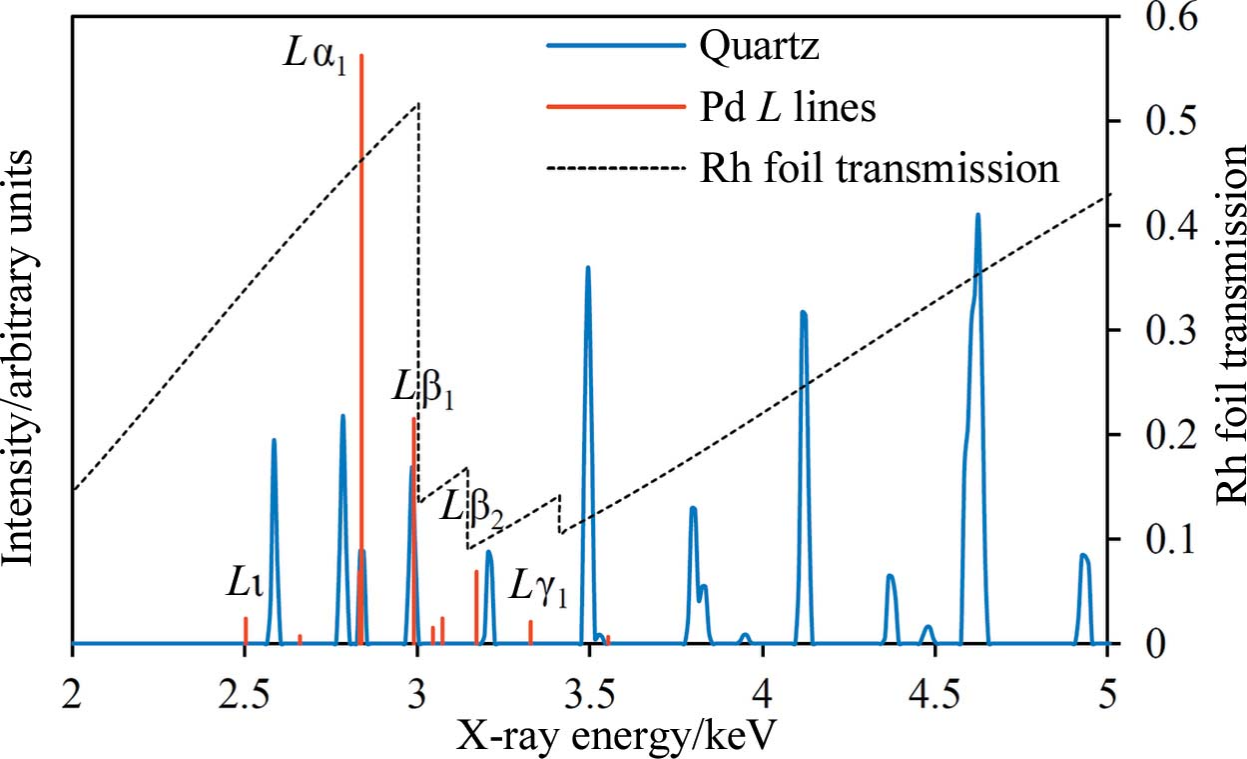
The simulated EDXRD spectrum of quartz at 2θ = 154.5°, along with the positions and relative intensities of the Pd *L* characteristic emission lines and the calculated transmission curve of a 1 µm Rh foil mounted on a 7 µm polyester support (right-hand scale). The EDXRD spectrum incident at the detector is shown, prior to detection. Only the more intense Pd *L* emission lines are labelled.

It is possible to increase the intensity of the enhanced diffraction peaks further, relative to other peaks in the spectrum (whether due to fluorescence or diffraction), by incorporating a suitable foil into the X-ray beam incident on the sample or the diffracted beam. The principle of this method is illustrated in Fig. 2 ▸ for the quartz–Pd coincidence listed in Table 1 ▸. The figure shows a simulated (Hansford, 2009 ▸) energy-dispersive XRD spectrum of quartz at the relevant 2θ angle overlaid by the *L*-series emission lines of Pd. Also shown in the figure is the calculated (Henke *et al.*, 1993 ▸) X-ray transmission curve of a 1 µm Rh foil mounted on a 7 µm polyester support (available from Goodfellow Cambridge Ltd, Huntingdon, UK). Fortuitously, the Rh *L* ^III^ absorption edge occurs at 3004.0 eV, just above the Pd *L*β_1_ line. Incorporating an Rh foil into the incident or diffracted beam will suppress spectral features immediately above and, to a lesser extent, below the quartz–Pd coincidence lines. The Rh foil serves to isolate the coincidence signals partially from other peaks in the spectrum. Note that the EDXRD spectrum shown in Fig. 2 ▸ is prior to detection by the X-ray detector, and illustrates the physical interactions taking place within what is effectively a high-spectral-resolution domain. The spectral resolution is lowered only at the final step, *viz.* the detection of the X-rays by the energy-resolving detector.

For the austenite–InTi coincidence, a Ti foil will serve a similar purpose. The Ti *K* absorption edge at 4964.9 eV lies above the Ti *K*β transition (as it must) by 33.1 eV. Modelling suggests that a 10 µm foil will be effective in enhancing this coincidence.

## 3. Experimental conditions   

The experimental setup for the results presented in this paper was very similar to those reported by Hansford (2013 ▸) and Hansford *et al.* (2014 ▸). This setup employs an in-house X-ray tube with a Cu anode and an e2v CCD-22 (Burrows *et al.*, 2005 ▸) charge-coupled detector, which has an open-electrode structure for enhanced low-energy quantum efficiency. Although an imaging detector is not, in general, required to implement the phase-targeted XRD technique, it is nevertheless useful to use one while developing the methodology. The CCD has an imaging area of 600 × 600 pixels with a 40 µm pixel width. It was cooled to 183 K using liquid nitrogen and operated in a photon-counting frame-transfer mode using in-house control electronics. An FWHM spectral resolution of 195 eV was achieved at an X-ray energy of 5.9 keV. All the spectra reported here were derived by selecting single-pixel CCD events only (Owens *et al.*, 1994 ▸). The quantum efficiency of the CCD exceeded 0.55 in the range 1.5–5 keV for single-pixel events.

The source materials used to generate the desired characteristic emission lines were available as 0.5 mm thick plates. These were simply attached to the emitting surface of the copper anode with two retaining screws. The plate must have a sufficiently good contact with the copper to allow electrical and thermal conduction, but it has been found that no special measures are required to ensure this.

The data acquisition times were 5 h, necessitated by the relatively long X-ray path lengths in the experimental setup, unless otherwise noted in the figure captions. In order to delineate the enhancement of the coincidence diffraction peaks, spectra were extracted from ‘on-coincidence’ and ‘off-coincidence’ regions of the CCD, equal in area. For some data sets, an attenuating filter was mounted either in the X-ray beam between the source and sample or in front of the CCD; full details are given with the description of each data set. These filters were used in addition to the polyimide/Al filter reported by Hansford *et al.* (2014 ▸). The aperture between the source and sample was either 2 or 3 mm in diameter for the data sets reported here.

To facilitate the measurement of austenite in steel samples, two methods of generating In and Ti characteristic X-rays simultaneously were attempted. Firstly, small plates of 99.6+% Ti and 99.999% In (both from Goodfellow) were butted together on the anode surface, positioned so that the join lay beneath the electron impact spot, which is typically 2–3 mm in diameter. Trial and error were used to find a good position for the join so that the two enhanced austenite diffraction peaks had approximately equal intensities. Secondly, a thin layer of In was electrodeposited onto Ti plates. The plates were degreased in Anapol Cleaner C, followed by a de-ionized water and acetone rinse. In was deposited in a Hull cell from a proprietary ethylene glycol-based solution of 0.87 *M* InCl_3_ (rights owned by the University of Leicester), which was heated to 323 K. An anodic etch was performed at 18 V for 5 min, followed by plating at 18 V for 10–15 min. The plates were then removed and rinsed in de-ionized water and acetone. A triangular arrangement of the electrodes was used to vary the thickness of the In layer along the length of the Ti plates. In addition, the deposition time was varied by trial and error in order to achieve a suitable thickness of In, with the criterion of approximately equal enhanced diffraction peaks. No attempt was made to measure the thickness of In on suitable samples, but it was estimated to be a few micrometres.

Proof-of-principle data were acquired using both of these methods. However, neither method proved to be entirely satisfactory. In the first method, the In plate appeared to suffer partial melting at the electron impact spot and the intensity of the In-enhanced diffraction peak dropped by several percent over the course of a few hours. Pure In has a relatively low melting point of 429 K. The In-plated Ti proved to be more robust, but nevertheless the In signal suffered a similar decrease over several days of use. Future attempts will focus on production of an alloy of In and Ti (Gulay & Schuster, 2003 ▸) which, it is hoped, will serve to stabilize In under electron impact.

## 4. Results   

### 4.1. Quartz–Pd coincidence   

**Figure 3:**
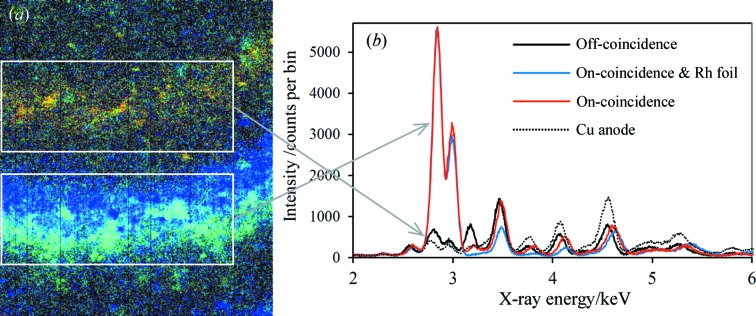
(*a*) A CCD image of the quartz diffraction pattern (source excitation voltage 8 kV, emission current 0.75 mA), energy-selected in the range of approximately 2.75–3.3 keV. X-ray energies within this range are represented by different colours. The two boxed regions indicate the areas from which the on-coincidence (bottom) and off-coincidence (top) spectra were extracted. (*b*) The extracted spectra from the on- and off-coincidence regions shown in part (*a*). Also shown are the on-coincidence spectrum with a 1 µm Rh foil in the X-ray beam incident on the sample (source 8 kV, 1.0 mA) and the spectrum acquired with no Pd plate on the Cu anode (source 8 kV, 0.45 mA), extracted from the off-coincidence region shown in part (*a*). The on-coincidence spectrum with the Rh foil was normalized to the Pd *L*α_1_ peak of the on-coincidence spectrum without the foil. The Cu anode spectrum was scaled so that the diffraction peak at ∼2.6 keV is equal in intensity to the corresponding peak in the off-coincidence spectrum. For all four data sets, the peak near 3.5 keV is mainly due to the pile-up of Si *K* fluorescence events.

In order to demonstrate experimentally the principle of phase-targeted XRD in the simplest possible configuration, a pressed-powder pellet of quartz was mounted in the vacuum chamber and a Pd plate attached to the X-ray tube anode. The CCD was positioned to encompass approximately 2θ = 148–168°; the CCD image and extracted spectra are shown in Fig. 3 ▸. The image shows arcs of three diffraction rings, two of which are overlapped and are the quartz–Pd coincidence lines listed in Table 1 ▸. The diffraction arcs are relatively ‘spotty’ because of the moderately large crystallite sizes in the sample (sieved to <75 µm). Each diffraction arc has a breadth of about 2.9° 2θ because of the modest incident-beam collimation employed in the experimental setup. The third, weaker, diffraction arc is due to diffraction of the Pd *L*β_2_ line at 3171.8 eV by quartz 201. The two boxed regions on the CCD image show the equal-area on- and off-coincidence regions from which X-ray spectra were extracted for Fig. 3 ▸(*b*). Comparison of these two spectra clearly shows the enhancement of the diffraction signal at the Pd *L*α_1_ and Pd *L*β_1_ energies, each by a factor of approximately 10. Also shown in Fig. 3 ▸(*b*) is the equivalent spectrum acquired with the Cu anode, which shows that there is some enhancement at the Pd *L*α_1_ and Pd *L*β_1_ energies, even in the off-coincidence spectrum. This enhancement can be explained by Rayleigh scattering, which serves to scatter the X-ray spectrum incident on the sample towards the detector with a modified intensity profile and will occur to some degree for all samples. In this instance, the effect is only noticeable at the more intense Pd *L* characteristic energies. Lastly, Fig. 3 ▸(*b*) shows the on-coincidence spectrum acquired with a 1 µm Rh foil in the incident beam. This spectrum shows that the foil is effective in suppressing the diffraction peaks immediately above the coincidence energies. The effect is not dramatic for a pure quartz sample but is expected to be more effective for real samples, especially those exhibiting K and/or Ca fluorescence peaks. The Rh foil was available only as a 10 mm diameter disc, which could readily be mounted in the incident beam but, unfortunately, not in the diffracted beam, because the CCD is significantly larger.

**Figure 4:**
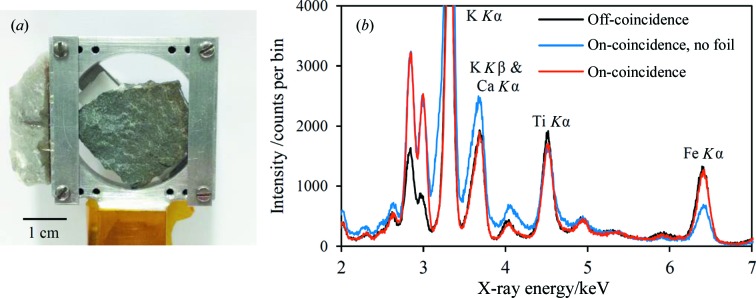
(*a*) A photograph of the mudstone rock sample mounted in the sample holder, with a scale bar. (*b*) The quartz–Pd off- and on-coincidence spectra, using the same CCD regions as shown in Fig. 3 ▸. The on-coincidence spectra both with (source excitation 8 kV, 1.0 mA) and without (8 kV, 0.35 mA) the Rh foil in the incident beam are shown, illustrating especially the suppression of the K *K*α fluorescence peak. The on-coincidence spectrum without the foil has been normalized to the Pd *L*α_1_ and Pd *L*β_1_ peaks (*i.e.* the coincidence peaks) of the corresponding spectrum with the Rh foil.

A representative geological sample is shown mounted on the sample holder in Fig. 4 ▸(*a*), along with X-ray spectra in Fig. 4 ▸(*b*). The sample is an unprepared mudstone rock known to contain quartz, along with chlorite, mica, plagioclase and potassium feldspars, and minor magnetite. The relative amounts of the minerals have not been quantified, but qualitative assessment of Bruker D8 Advance data suggests quartz is present in the tens of wt% range. The Rh foil was mounted in the X-ray beam incident on the sample. The spectra extracted from the on- and off-coincidence regions of the CCD (Fig. 4 ▸ *b*) reveal a clear enhancement of the diffraction intensity at the Pd *L* coincidence energies. The Pd *L*α_1_ peak also appears to be enhanced in the off-coincidence spectrum, probably because of an interfering phase. Also shown on the graph is the on-coincidence spectrum with no foil in either the incident or diffracted beam, illustrating the suppression of K fluorescence and the resulting isolation of the coincidence signal. Inclusion of the Rh foil serves to reduce the background signal substantially in the vicinity of the Pd *L*α_1_ and Pd *L*β_1_ energies.

**Figure 5:**
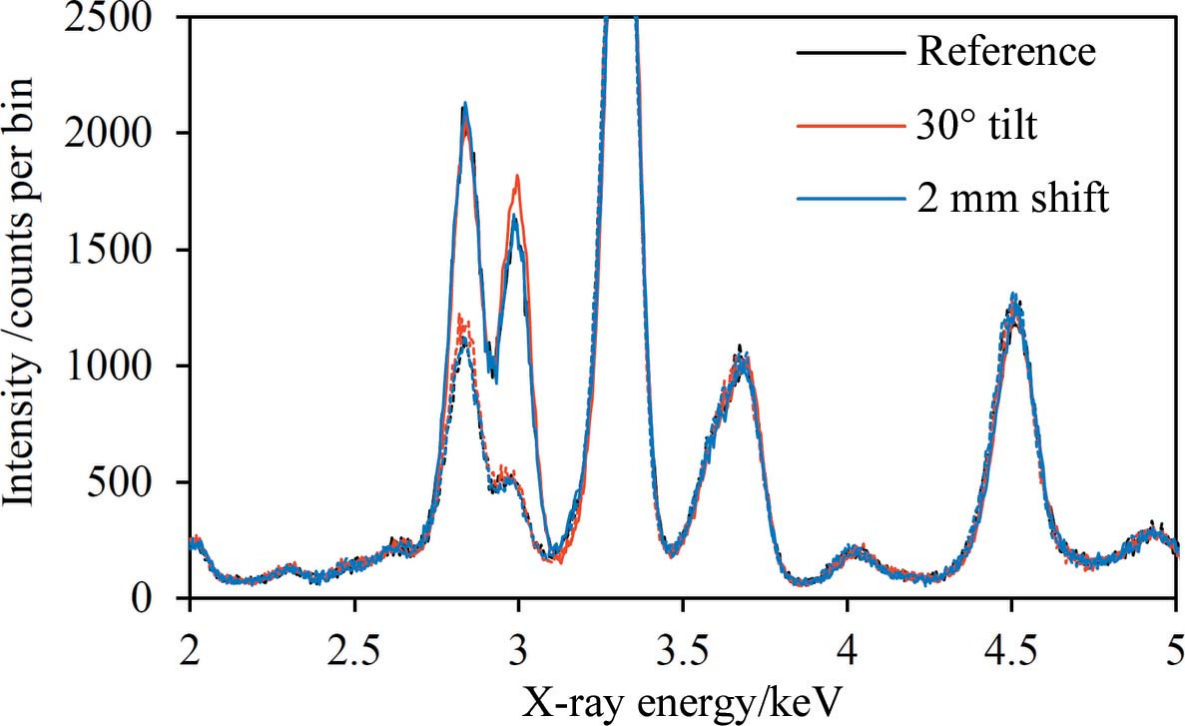
Quartz–Pd on- and off-coincidence spectra acquired using the mudstone rock sample shown in Fig. 4 ▸(*a*) mounted in three different positions: a reference position, rotated by 30° about the axis passing through the analysed surface of the sample and perpendicular to the plane of the experiment, and shifted by 2 mm away from the X-ray source. The source excitation conditions were 8 kV and 0.45, 0.38 and 0.47 mA, respectively. The on- and off-coincidence spectra are shown as solid and dashed lines, respectively. The spectra have been scaled to have equal K *K*α fluorescence peak intensities. Each data set was acquired over 3 h with the Rh foil mounted in the incident beam.

The results shown in Fig. 4 ▸ demonstrate the feasibility of the phase-targeted XRD method for unprepared samples exhibiting significant surface morphology. To illustrate this feature of the technique further, data were acquired for the same sample rotated through 30° and moved 2 mm away from the source and detector. The quartz Pd *L*α_1_ and Pd *L*β_1_ diffraction arcs were located by inspection on the CCD images, and they showed small shifts in position of approximately Δ(2θ) = 0.5° because of the change in sample position relative to the reference position. The same on- and off-coincidence regions were selected for the three data sets and the extracted spectra are shown in Fig. 5 ▸. There is very good correspondence between both the on- and off-coincidence spectra for the different sample positions, further demonstrating the applicability of the method to real unprepared or minimally prepared samples.

### 4.2. Austenite–InTi coincidence   

**Figure 6:**
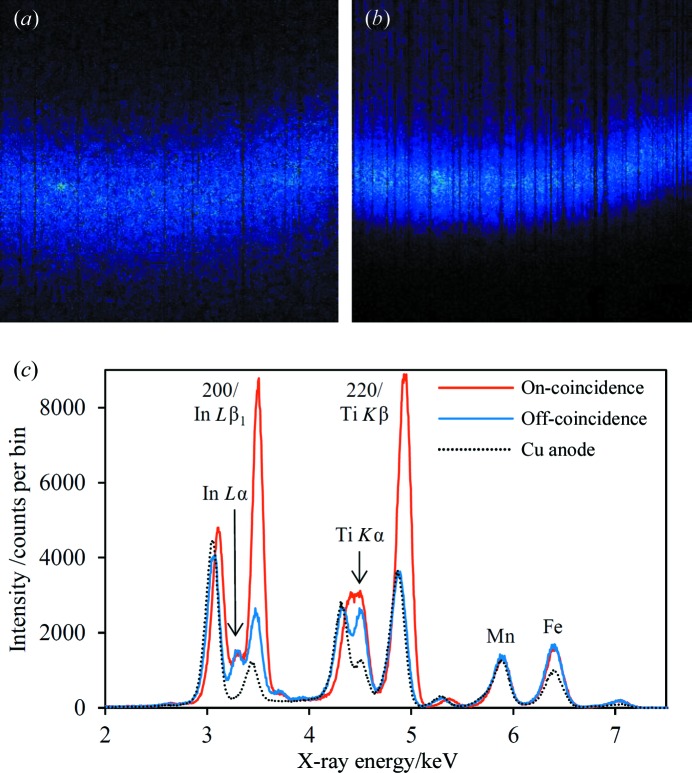
Results acquired for the TWIP steel sample, with a 10 µm Ti foil mounted in front of the CCD; source excitation was 7.5 kV with an emission current of 1.0 mA. (*a*) A CCD image of the In *L*β_1_/austenite 200 diffraction arc. (*b*) A CCD image of the Ti *K*β/austenite 220 diffraction arc. (*c*) The on- and off-coincidence spectra extracted from the CCD data. The selected off-coincidence region was at the top of the CCD. The spectrum acquired with a Cu anode (source 7.5 kV, 1.4 mA; taken from the off-coincidence region of the CCD) is also shown for comparison, approximately normalized in intensity over the 4–6 keV range with the off-coincidence spectrum. Along with the enhanced diffraction peaks, the more prominent scattered and fluorescence peaks are labelled.

The coincidence XRD results presented in this subsection were all acquired using Ti plates coated with In, as described in §3 and subsequently referred to as an InTi source. Samples of several different types of steel have been used to test the coincidence XRD method, including an austenitic high-Mn TWIP (twinning-induced plasticity) steel, a martensitic mild-carbon steel containing boron, and several dual-phase low-carbon steels that have undergone different heat treatments. The results for the TWIP steel sample are shown in Fig. 6 ▸, including images of the In *L*β_1_ and Ti *K*β diffraction arcs in Figs. 6 ▸(*a*) and 6 ▸(*b*), respectively. Details of the diffraction arcs are discussed in §4.2.1. The extracted spectra are shown in Fig. 6 ▸(*c*) for on- and off-coincidence regions, and also for a data set acquired without the InTi plate mounted on the Cu anode. The main fluorescence and scattering peaks have been labelled, including the two coincidence diffraction peaks, which are the strongest peaks in the on-coincidence spectrum. The enhancement factors are approximately 4.9 and 2.7 (after subtracting the background) for the 200 and 220 austenite diffraction peaks, respectively. Relative to the Cu anode 200 peak, the enhancement factor is ∼7.0. The 200 peak is significantly weaker than 220 in the Cu anode data set, illustrating how the two enhanced peaks can be equalized in intensity by optimizing the thickness of the In layer on the InTi source. It should be noted that the Ti *K*α peak is due to fluorescence from the Ti foil in front of the CCD, as there is no Ti in the sample. There will be a small contribution to the Ti *K*β peak in all three data sets arising in the same way. The amount of austenite in this sample is not known accurately, but is estimated to be roughly 40% from the data presented here. Fits to the spectra suggest that the balance is dominated by martensite rather than ferrite.

**Figure 7:**
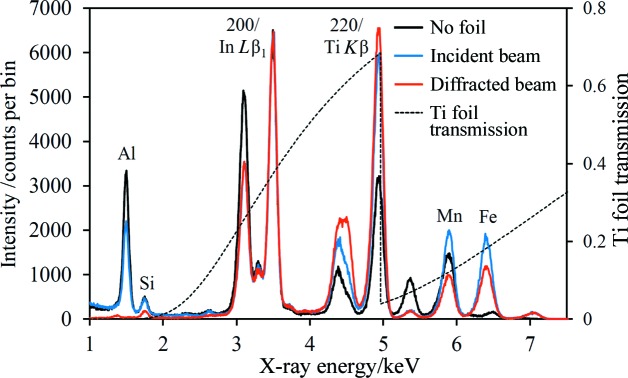
On-coincidence spectra for the TWIP steel sample with no foil and with a 10 µm Ti foil mounted in the incident and diffracted X-ray beams (see main text for source excitation conditions). The three spectra have been normalized to the intensity of the In *L*β_1_/austenite 200 peak. The main fluorescence peaks have been labelled, along with the coincidence peaks. Also shown is the calculated transmission curve of the 10 µm Ti foil (right-hand scale).

The effect of the 10 µm Ti foil was investigated by acquiring data with no foil, and with the foil mounted in the incident and diffracted X-ray beams; Fig. 7 ▸ shows the resulting on-coincidence spectra for the same TWIP steel, along with the transmission curve of 10 µm Ti (Henke *et al.*, 1993 ▸). The data set with no foil was acquired with a source voltage of 7 kV (and emission current 0.4 mA) in order to avoid stimulating Fe *K* fluorescence, which would be very strong with a higher voltage. This spectrum serves as a point of reference for the other two. When the foil is mounted in either the incident or the diffracted beam, the source voltage can be increased because Fe *K* (and Mn *K*) fluorescence is suppressed quite efficiently. A source voltage of 7.5 kV was used when acquiring these two data sets, with emission currents of 0.75 and 1.0 mA, respectively. When interpreting these spectra, it is important to note the differing effects of the attenuating foil on diffraction, Rayleigh scattering and fluorescence peaks, which are a consequence of the fundamental differences in the physical processes. Diffraction and Rayleigh scattering are elastic processes, so the foil transmission curve directly predicts the decrease in intensity that can be expected upon insertion of the foil into either the incident or the diffracted beam, assuming all other conditions remain the same. Fluorescence is stimulated by all X-rays with energy greater than the corresponding elemental absorption edge. It follows that there can be a considerable difference in the effect on fluorescence peaks, depending on whether the foil attenuates the incident or diffracted beam. The difference can be seen most clearly in the magnitude of the Al fluorescence peak in Fig. 7 ▸. When the foil is in the incident beam, Al fluorescence can be stimulated quite efficiently by essentially all the X-ray photons reaching the sample, and so the Al peak is only a little less intense than in the reference spectrum. Conversely, the transmission of 1.5 keV X-rays through the foil when it is mounted before the detector is negligible. Similar arguments apply to the Mn *K* and Fe *K* fluorescence peaks, although the effect is much less marked. It can be seen from the spectra that the foil is most effective when positioned in front of the detector, but the improvement relative to incident beam attenuation is modest. As a result of the overall attenuating effect of the foil mounted in either position, a higher source voltage and/or current is required in order to achieve signal levels comparable to the data with no foil used.

**Figure 8:**
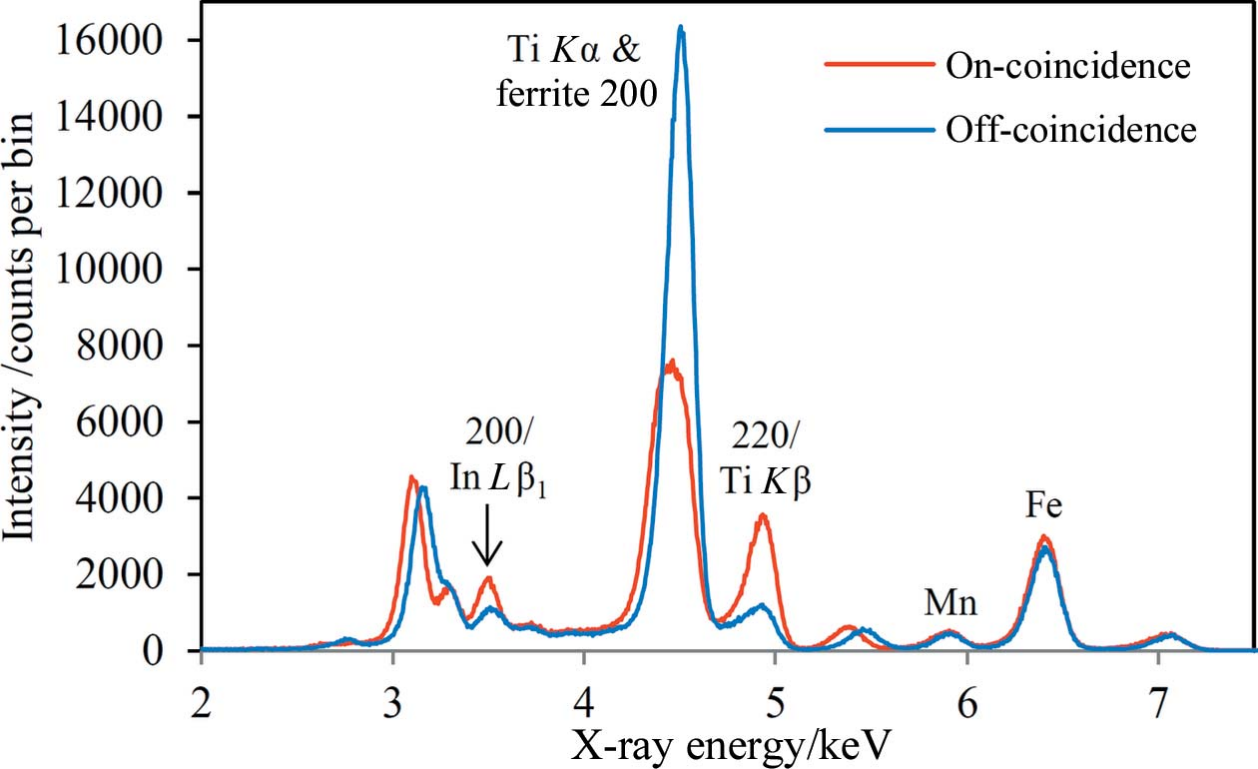
The on- and off-coincidence spectra for a dual-phase steel sample with a 10 µm Ti foil mounted in front of the CCD; source excitation 7.5 kV, 1.2 mA. The off-coincidence region on the CCD includes part of a diffraction arc that arises from diffraction of Ti *K*α radiation by ferrite 200, leading to enhancement of this peak in the off-coincidence spectrum.

To test the austenite–InTi coincidence method at lower austenite levels, several of the dual-phase steel samples were mounted in the experimental chamber with the 10 µm Ti foil mounted in front of the CCD. The coincidence XRD results are shown in Fig. 8 ▸ for one of these samples. The phase composition of this sample has been measured using conventional XRD methods by Tata Steel with the following results: austenite 6.8 wt%, ferrite 85.4 wt%, martensite 7.7 wt%. The coincidence diffraction arcs for this sample lie at 2θ = 160.0°, higher on the CCD than for the TWIP steel sample. The off-coincidence region has therefore been taken towards the bottom of the CCD, with the consequence that the diffraction peaks are shifted in the opposite direction relative to the on-coincidence peaks compared with the data in Fig. 6 ▸. The expected enhancement of the austenite 200 and 220 diffraction peaks is observed, with enhancement factors of 2.1 and 3.4, respectively. The enhancement of the 200 peak is lower than for the TWIP sample, whereas the 220 enhancement is greater. This result is attributed to the degradation of the In layer on the InTi source plate during electron impact – the dual-phase sample data were taken later than the TWIP sample data, after ∼30 h use of the InTi source. Nevertheless, the results show a useful enhancement of these two austenite peaks at relatively low levels of austenite.

#### 4.2.1. Austenite–InTi diffraction arcs   

Two points are immediately noticeable from the CCD images in Figs. 6 ▸(*a*) and 6 ▸(*b*): the Ti *K*β diffraction arc is significantly sharper (less spread in 2θ) and occurs at a higher scattering angle than the In *L*β_1_ diffraction arc. On the basis of the calculation in Table 1 ▸, it was expected that the two diffraction arcs would align accurately with each other. Four of the dual-phase steels tested had significant austenite, allowing comparison with the TWIP steel results. Two of the dual-phase steels had broader In *L*β_1_ diffraction arcs, whereas for the remaining two the In *L*β_1_ and Ti *K*β diffraction arcs were comparable in width. The observed ranges of widths were 5.0–7.3° (FWHM) for the In *L*β_1_ diffraction arc and 3.5–6.1° for Ti *K*β. Monte Carlo ray tracing (Hansford, 2009 ▸) predicts FWHM widths of ∼2.8° for each diffraction arc, assuming only geometric broadening with no contribution from, for example, crystallite size. The Ti *K*β diffraction arc was consistently found to occur at a higher scattering angle, with the difference ranging from 0.8° for the TWIP steel to 1.1–1.6° for the dual-phase steels (determined with ±0.1° accuracy). The 2θ values for the Ti *K*β diffraction arc varied between 156.4 and 161.0° across the five samples.

A possible explanation of the variation in the widths of the diffraction arcs lies in the different penetration depths of In *L*β_1_ and Ti *K*β X-rays; the 1/e penetration depths into pure iron (taken as an approximation of the steel composition) are calculated to be 3.4 and 8.7 µm, respectively, and the two X-ray energies therefore sample significantly different sets of crystallites. If the average crystallite size is smaller towards the surface of the steel, the In *L*β_1_ diffraction arc would be preferentially broadened.

Regarding the scattering angles, several possible explanations have been considered for the consistent offset between the two diffraction arcs, albeit with some sample-to-sample variation in magnitude, as follows:

(i) The effect of energy-dependent sample penetration depths on the apparent scattering angles.

(ii) Separation of the source positions for the In *L* and Ti *K* characteristic emission lines.

(iii) A discrepancy in the *d*-spacing ratio relative to the calculation.

(iv) A discrepancy in the characteristic energy ratio relative to the calculation.

These four possibilities are considered in turn. Calculation of the effect of the energy-dependent penetration depths on the apparent 2θ scattering angle, using the experimental geometry, gives an apparent error of only 0.002°, much too small to provide an explanation. The second possibility seems unlikely when the InTi source consists of an In layer deposited on a Ti plate – this layer is expected to be uniform and there are no visual indications of inhomogeneity on the scale of a few millimetres. Also, the offset between the diffraction arcs did not change significantly when different InTi plates were substituted as the source. If the In and Ti source positions were separated by 1 mm in the equatorial plane (*i.e.* the plane in which the source, sample and detector lie), the effect on the apparent scattering angle is calculated to be 0.15°, which does not explain the magnitude of the observed values. Interestingly, one of the five samples with significant austenite was tested with Ti and In plates butted up to each other on the X-ray source anode as well as with an In layer on a Ti plate. The angular separation of the diffraction arcs increased by 0.2° when the butted plates were used, suggesting a separation of 1.3 mm in the average source locations of the In *L* and Ti *K* characteristic emission lines, which is reasonable given the 2–3 mm source spot size.

Regarding the third possibility listed above, the calculated *d*-spacing ratio is, in a simple analysis, exact for a cubic phase (§2.1). However, the sample-to-sample variation in the offset between the diffraction arcs is direct evidence for variation in the *d*-spacing ratios. A plausible explanation for the diffraction peak shifts is the effect of stacking faults on 111 planes (Blawert *et al.*, 2001 ▸). Using equation (1) of Blawert *et al.* (2001 ▸) and assuming a stacking fault probability of α = 0.02 determined for untreated (non-expanded) austenite in that paper, the calculated shifts in the 200/In *L*β_1_ and 220/Ti *K*β peaks are Δ(2θ) = −0.74 and 0.37°, respectively, giving an offset between these two peaks of 1.11° (assuming a zero offset for an undistorted crystal structure). The magnitude of this offset for the assumed stacking fault density is consistent with the observations presented here, and the sign of the offset also agrees. Additionally, Warren (1969 ▸) shows that stacking faults are also expected to have an effect on peak widths, with greater broadening expected for the 200 peak on the basis of stacking and twin faulting probabilities. The results presented here are again consistent with this expectation.

It is worth considering also the fourth possibility listed above. The characteristic emission line energies given by Deslattes *et al.* (2003 ▸) are *E*(In *L*β_1_) = 3487.244 (58) eV and *E*(Ti *K*β) = 4931.827 (59) eV, which gives a ratio of 1.414248 (40). However, the Ti *K*β line is composed of two closely spaced transitions, *K*β_1_ and *K*β_3_, the former expected to be about twice as intense as the latter. Theoretical calculations predict a difference in energy between these transitions of 3.6 eV (Deslattes *et al.*, 2003 ▸), which will result in a broadening of the Ti *K*β diffraction arc of ∼0.5° for the conditions described in Table 1 ▸, a relatively minor contribution to the observed widths. The In *L* series of emission lines has a theoretical *L*_2_ *M*_5_ line at 3494.8 eV; however, this transition violates the quantum number selection rules for electric dipole and quadrupole transitions and magnetic dipole transitions (Markowicz, 2002 ▸), so is expected to be extremely weak.

## 5. Discussion   

The phase-targeted XRD technique described in this paper identifies microcrystalline phases *via* the uniqueness of their *d* spacings. Whether the detection of two or more *d* spacings present in a sample translates into phase specificity depends on a number of factors, including the accuracy of the exploited coincidence of *d*-spacing and characteristic energy ratios, and the angular resolution afforded by the experimental configuration. These factors result in the detection of (small) *d*-spacing ranges rather than highly precise *d* spacings *per se*. An important factor is the nature of the intended application. Some applications will essentially guarantee that there are no interfering phases, and the detection of austenite in steels is an example. The other major phases present, ferrite and martensite, do not have any *d* spacings close to the enhanced austenite *d* spacings, and the only possible interferences are from iron carbides, which are present at quite low levels. Detection of a targeted phase in geological samples is more likely to suffer interferences because of the complex mineral assemblages common in geology. Nevertheless, some mining applications may be feasible because the number of minerals present is often more limited and their identities are known in advance. Detailed knowledge of the application is likely to be crucial in the development of phase-targeted XRD for any potential application.

The philosophy underlying the phase-targeted XRD technique is that it enables the detection and quantification of target phases more sensitively than otherwise possible in a simplified fixed-geometry and low-cost instrument format. It is envisaged that purpose-built instruments would have a compact design with X-ray path lengths of the order of a few centimetres at most, comparable to handheld X-ray fluorescence devices and a proposed handheld XRD instrument (Hansford, 2011 ▸). A compact design avoids the need for a vacuum despite the low X-ray energies, and is expected to shorten measurement times to a few minutes.

Two important factors in phase-targeted XRD are the stability and predictability of the *d* spacings of the target phase. For example, quartz is known to exhibit a small natural variation in its unit-cell dimensions and therefore its *d* spacings (Moore & Reynolds, 1997 ▸), so the angular positions of the coincidence diffraction rings given in Table 1 ▸ should also vary very little. In contrast, the unit-cell size of austenite is dependent on the proportion of alloying elements in the steel and, additionally, the *d* spacings are affected by the stacking and twin faulting probabilities. Use of an imaging detector can overcome these issues and permit the extraction of more detailed information about the sample, but also represents a move away from the simplicity of the basic phase-targeted XRD idea. It is worth noting that, for in-line production of specific types of steel, the carbon content and other properties of the target austenite phase might be narrowly defined, opening again the opportunity to use a non-imaging detector in a fixed geometry.

As already noted in §4.2.1, the use of low-energy X-rays means that penetration depths are of the order of a few micrometres. For geological samples, it is likely to be advantageous to measure freshly cleaved surfaces in order to avoid weathering rinds, for example. For metallurgy applications, the phase-targeted XRD method is essentially a surface measurement technique. It can be expected that phase quantification will be representative of the bulk, as assumed, for example, in quantitative metallography. Any surface layer gradients in composition, residual stress and fault densities will serve to broaden the diffraction peaks, but such effects are essentially secondary.

The experimental diffraction peak enhancement factors for austenite–InTi are in the range 2.1–4.9, significantly lower than the enhancement factors of ∼10 achieved for quartz–Pd. One disadvantage of using a binary-element anode is the dilution of each of the two elements relative to the pure element, leading to lower intensities of the emitted characteristic X-rays. Also, the austenite–InTi coincidence involves emission lines that are not the strongest ones in the series.

Although the purpose of this paper is primarily to demonstrate the principle and feasibility of the method, the phase-targeted XRD technique can be expected to be quantitative. As a result of Rayleigh scattering of characteristic X-rays from the source, the main difficulty in quantification is likely to be accurately establishing the signal level in the absence of the targeted phase; this was the purpose of comparing the on- and off-coincidence signals when acquiring the data presented here. The off-coincidence scattering angle should be chosen to avoid any diffraction peaks arising from the characteristic energies that form part of the coincidence signal. Once again, knowledge of the intended application is crucial. An alternative is to perform a measurement at the coincidence angle on a sample that is as similar as possible to the sample(s) of interest but which does not contain the targeted phase. A calibration curve can be constructed using samples with known amounts of the target phase. The ultimate sensitivity of the technique depends on multiple factors and is another parameter that is highly application dependent. The results shown in Fig. 8 ▸ for 6.8 wt% austenite give reasonable hope that the measurement can be sensitive to retained austenite at the ∼1% level.

As long as the relative intensities of the X-ray source characteristic lines are stable, the relative intensities of the enhanced diffraction peaks should be constant as long as three conditions are met:

(i) no interfering phases;

(ii) sampling of a sufficient number of crystallites to satisfy particle statistics requirements; and

(iii) absence of preferred orientation.

For applications in which the first two of these conditions are essentially guaranteed, the relative peak intensities can be used to extract simple texture information about the targeted phase, alongside quantification. Independent knowledge of the planes that are preferentially aligned with the sample surface is necessary in order to interpret the relative intensities correctly. The measurement of austenite in steels is again an example where these conditions are likely to be met in practice; the simultaneous quantification of the amount and texture of austenite is highly desirable in steel production.

The use of an elemental foil to isolate the coincidence signals to some degree from other peaks in the acquired spectrum is a generally applicable strategy in phase-targeted XRD. The foil should have an absorption edge (*K* and *L* ^III^ edges give the highest transmission contrast) as close as possible to, but higher than, the highest energy of the coincidence signal. The Rh foil used in the quartz–Pd coincidence is very good in this regard, with a separation of only 13.7 eV between the Rh *L* ^III^ edge and the Pd *L*β_1_ transition. For the austenite–InTi co­incidence, the Ti *K* absorption edge lies 33.1 eV above the Ti *K*β transition, which is still usefully close. The main dis­advantage of this foil is the emission of Ti *K* fluorescence from the foil, which gives a small signal interfering with the co­incidence signal. Unfortunately, there are no other practical elemental absorption edges that are close to the Ti *K*β transition.

Future work on the development of the phase-targeted XRD method will focus on testing quantification of the target phase. Establishing a good zero signal level is crucial to quantification, and it is preferable to achieve this by measurement of a sample known to have a negligible amount of the target phase. The alternative method of measuring on- and off-coincidence signals, as used for the data presented in this paper, has some disadvantages. Firstly, this method necessitates the use of two detectors instead of one, or an imaging detector, or requires movement of the detector. Secondly, interferences are more difficult to avoid because either of the on- and off-coincidence measurements can be affected by an interfering phase. For the austenite application, future work will also focus on establishing a more stable mixed InTi source. Alternative methods of production include co-melting of the two components and co-sputtering onto a substrate. Both these methods will give a more intimate mixture of In and Ti at the atomic level, possibly serving to stabilize the In under electron bombardment. Further work on the austenite application is also required to establish firmly the reasons for the angular separation of the coincidence diffraction arcs and their differing widths.

## Figures and Tables

**Table 1:** Selected coincidences for quartz and austenite phases

Phase	*d* spacings (Å) and Miller indices	*d*-spacing ratio	Characteristic emission lines	Energy ratio	Relative intensity†	Scattering angle and mismatch
Quartz‡	2.2366, (111)	1.0512	Pd *L*α_1_, 2838.6 eV	1.0534	3.23	2θ = 154.53°
	2.1277, (200)		Pd *L*β_1_, 2990.3 eV		2.66	2δ = 1.08°
Quartz‡	1.2879, (104)	1.0878	Ce *L*α_1_, 4840.1 eV	1.0874	1.78	2θ = 168.17°
	1.1840, (114)		Ce *L*β_1_, 5262.9 eV		1.23	2δ = 0.41°
Austenite§	1.8055, (200)	1.4142	In *L*β_1_, 3487.2 eV	1.4143	11.5	2θ = 159.86°
	1.2767, (220)		Ti *K*β, 4931.8 eV		6.26	2δ = 0.02°

† Intensities are approximate values on an arbitrary scale, taking into account both the X-ray emission line intensity and diffraction intensity.

‡ *d* spacings calculated from Smith & Alexander (1963 ▸).

§ For 1.0 wt% C; *d* spacings are calculated using the equations given in the text.
